# Bufei Huoxue capsule attenuates COPD-related inflammation and regulates intestinal microflora, metabolites

**DOI:** 10.3389/fphar.2024.1270661

**Published:** 2024-04-09

**Authors:** Yuanyuan Li, Jiali Chen, Yue Xing, Jian Wang, Qiuling Liang, Jiamin Zeng, Siyi Wang, Qiong Yang, Jianing Lu, Jieying Hu, Wenju Lu

**Affiliations:** ^1^ Guangzhou Medicine University,Guangzhou Institute of Respiratory Health, The First Affiliated Hospital of Guangzhou Medical, Guangzhou, China; ^2^ Guangzhou University of Chinese Medicine, Guangzhou, China; ^3^ First Affiliated Hospital of Guangzhou Medical University, Guangzhou, China; ^4^ Key Laboratory of National Health Commission for the Diagnosis and Treatment of COPD, Inner Mongolia People’s Hospital, Hohhot, China

**Keywords:** Bufei Huoxue capsule, chronic obstructive pulmonary disease, inflammation, intestinal flora, metabolites

## Abstract

**Background:** Bufei Huoxue capsule (BFHX) is widely used for the clinical treatment of chronic obstructive pulmonary disease (COPD) in China.

**Objectives:** The aim of this study is to explore the effects on COPD and the underlying mechanism of BFHX.

**The process and methods:** In this study, we established a COPD mouse model through cigarette smoke (CS) exposure in combination with lipopolysaccharide (LPS) intratracheal instillation. Subsequently, BFHX was orally administrated to COPD mice, and their pulmonary function, lung pathology, and lung inflammation, including bronchoalveolar lavage fluid (BALF) cell count and classification and cytokines, were analyzed. In addition, the anti-oxidative stress ability of BFHX was detected by Western blotting, and the bacterial diversity, abundance, and fecal microbiome were examined using 16S rRNA sequencing technology.

**Outcome:** BFHX was shown to improve pulmonary function, suppress lung inflammation, decrease emphysema, and increase anti-oxidative stress, whereas 16S rRNA sequencing indicated that BFHX can dynamically regulate the diversity, composition, and distribution of the intestinal flora microbiome and regulate the lysine degradation and phenylalanine metabolism of COPD mice. These results highlight another treatment option for COPD and provide insights into the mechanism of BFHX.

## Introduction

Chronic obstructive pulmonary disease (COPD) is a heterogeneous lung disease that is characterized by chronic respiratory symptoms due to abnormalities of the airways (bronchitis and bronchiolitis) and/or alveoli (emphysema), which cause persistent, progressive airflow obstruction, and it is the third most common cause of death worldwide. As more than three-quarters of patients with COPD worldwide are from low-income and middle-income countries, it remains difficult to achieve a substantial reduction in the burden of COPD globally. Most patients with COPD suffer concomitant chronic comorbid diseases, and comorbidities directly impact on the risk of hospitalization and death (Miller et al., 2013). Treatment for COPD mainly includes abstinence from smoking, medication therapy, lung rehabilitation, oxygen therapy, and non-invasive ventilator support. Although these therapies can alleviate symptoms, reduce the frequency and severity of exacerbations, and improve health status and exercise tolerance to a certain extent, they largely fail to prevent the progression of COPD.

In recent years, mounting evidence has highlighted the role of the gut–lung axis in pathological conditions. The composition and metabolism of the microbiome influence the immune system and inflammation of the lung, whereas pulmonary disease indirectly affects the microbiome and metabolism of the gut, thus forming a vicious circle (Keely and Hansbro, 2014; Budden et al., 2017). Moreover, bowel inflammation can damage pulmonary function if left untreated, resulting in irreversible lung damage (Mateer et al., 2015; Raftery et al., 2020). Patients with COPD have a higher incidence of gastrointestinal disturbances than the general population, whereas gastrointestinal disease and ulcerative colitis are correlated with increased mortality in patients with asthma-related COPD (Ekbom et al., 2008; Mateer et al., 2015; Budden et al., 2017a; De Nuccio et al., 2022). The gut microbiome has also been shown to regulate inflammation in acute and chronic respiratory diseases, including COVID-19 and COPD (Budden et al., 2019; Bai et al., 2022).

Bufei Huoxue capsule (BFHX), which consists of *Astragalus mongholicus* Bunge, *Paeonia anomala subsp. veitchii* (Lynch), and *Cullen corylifolium* (L.) Medik, is widely used clinically to treat patients with COPD (Li et al., 2023). BFHX not only improves the respiratory symptoms and reduces the frequency of exacerbations in patients with COPD but also increases patients’ quality of life and exercise tolerance (Chen et al., 2022). Based on these findings, we sought to establish whether BFHX functions as a treatment for COPD by regulating the gut flora. We aimed to lay a scientific foundation for further understanding the pharmacological effect of BFHX on COPD and COPD-related flora disorders.

## Materials and methods

### BFHX components

The BFHX capsule consists of *Astragalus exscapus* L., *Paeonia lactiflora* Pall, *Psoralea aphylla* L., *Paeonia lactiflora* Pall, and *Psoralea aphylla* L. *Astragalus exscapus* L. is dry root of *Astragalus membranaceus* (Fisch.) Bge. var. *mongholicus* (Bge.) Hsiao and *Paeonia lactiflora* Pall is dry foot *of Paeonia lactiflora* Pall. In addition, *Psoralea aphylla* L. is dry and contains mature seeds of *Psoralea corylifolia* L.

Approximately 720 g of *Astragalus exscapus L.*, 540 g of *Paeonia lactiflora* Pall, and 360 g of *Psoralea aphylla L. were* boiled with 8 times the amount of water for the first time and 6 times the amount of water for the second time, for 1 h, respectively. The combined decoction was filtered and concentrated to a relative density of 1.05–1.15 (80°C); next, ethanol was added to the decoction to achieve a 60% alcohol content for 24 h, ethyl alcohol was recycled, and the supernatant was taken to concentrate to a relative density of 1.35–1.40 (80°C). A measure of 180 g of *Paeonia Lactiflora* Pall was ground to fine powder, and then the powder was mixed well with the supernatant and ground to fine powder. The precipitation was collected and washed with 90% ethanol in turn, and then it was dried routinely to obtain the powder.

BFHX preparation and quality control were conducted as described previously (Li et al., 2023). The C-18 column (150 mm × 4.6 mm, Agilent, Italy) was equipped with a guard column for BFHX capsules and standard solutions. Three mobile phases were conducted as follows: (1) an aqueous solution containing water/acetonitrile (v/v, 68:32) (*Astragalus exscapus* L.), (2) an aqueous solution containing 0.05 mol/L potassium dihydrogen phosphate/methanol (v/v, 60:40) (*Paeonia lactiflora* Pall), and (3) an aqueous solution containing water/methanol (v/v, 52:48) (*Psoralea aphylla* L.).

### Animal model and administration

Six- to eight-week-old male C57 mice were purchased from Charles River (Beijing, China) and kept under SPF conditions (12 h light/dark cycle) at a temperature of 24°C ± 2°C and 50%–60% humidity. Mice were provided water and food freely. The animal experiment was approved by the Animal Health Research Institute of Guangdong Academy of Agricultural Sciences (ethics no. SPF2020034). After a 1-week quarantine period, mice were randomly divided into the following groups: 1) control group (CTL), 2) COPD model group (MOD), 3) COPD + roflumilast (POS group, 0.5 mg/kg/d), 4) COPD + BFHX low-dose group (low group, 1.9 g/kg/d, based on the clinical dose), and 5) COPD + BFHX high-dose group (high group, 3.8 g/kg/d), n = 9 per group. All groups, except the CTL group, received cigarette smoke (CS) exposure and lipopolysaccharide (LPS) instillation, as described previously (Guan et al., 2020). Mouse airways were instilled with PBS or LPS (75 μg in 50 μL) on experimental days 1 and 14; all mice, except the CTL group, received CS exposure for 4 h with 4 h intervals. The animals were orally administered on the 61st day of the experiment for 30 days, the weights of the animals were recorded, and their states, including their hair status and movement, were observed. After administration, the animal was harvested for further analysis (n = 5–6 mice per group were analyzed).

### Pulmonary function

The small animal pulmonary function instruction-forced pulmonary maneuver system (DSI, CA, United States) was used to evaluate the pulmonary function of mice. Mice were anesthetized with 1.2% avertin (0.2 mL/10 g) by intraperitoneal injection. The trachea of each mouse was cannulated and placed in the body chamber, before the pulmonary function of mice was recorded. Lung function readings were excluded from the analysis after quality control.

### Bronchoalveolar lavage fluid (BALF) collection

BALF was collected by injection and aspiration of 0.8 mL of sterile saline solution repeated six times. The BALF was centrifuged at 800 rpm for 10 min at 4°C, and the supernatant was collected and stored at −80°C for cytokine assessment. The number of inflammatory cells in the BALF was counted, and the cell morphology was evaluated with hematoxylin–eosin (HE) staining. At least 200 cells on the slide were classified in a blinded manner.

### Lung histology

The left lungs of the mice were inflated with paraformaldehyde fixative and excised at a 20-cm H_2_O pressure. Subsequently, the lung tissues were embedded in paraffin, cut, dehydrated, and subjected to H&E staining. The mean linear intercept (MLI) of the lung tissue was measured. Periodic acid-Schiff (PAS) staining was performed to access the level of goblet cell hyperplasia, Masson’s staining was performed to evaluate collagen deposition, and α smooth muscle actin alpha (αSMA) IF staining was conducted to evaluate the expression level of the lung airway.

### Enzyme-linked immunosorbent assay (ELISA)

The concentrations of monocyte chemoattractant protein-1 (MCP1) and KC (functional IL-8 in mice) in the BALF supernatant were detected using ELISA kits, according to the respective manufacturer’s instructions. Briefly, the BALF supernatant was added to the ELISA plates pre-coated with capture antibodies and incubated overnight at 4°C. The ELISA plates were then washed with phosphate-buffered saline (PBS) and incubated with anti-detection antibodies at room temperature. Streptavidin-conjugated HRP was added to each well, followed by the enzyme-substrate TMB, and incubated for 10 min. The enzyme reaction was stopped by adding the 2N H_2_SO_4_ solution. The absorbance was read at 450 and 570 nm using a microplate reader (Gene, Hongkong, China). The concentrations of the investigated cytokines in the BALF were determined using standard curves.

### Western blotting assays

Frozen lungs were lysed in lysis buffer containing the protease inhibitor PMSF (100:1). Lysates were cleared by centrifugation at 4°C for 30 min, and the total protein concentration of tissues was determined using a BCA kit (Beyotime Biotechnology, Wuhan, China). Next, 10% SDS PAGE and Western blotting were performed using a standard method, and the proteins were transferred to a polyvinylidene difluoride (PVDF) membrane (Millipore, Billerica, MA) by Western blotting (Bio-Rad Laboratories, Hercules, CA). Membranes were blocked with 5% milk and incubated with primary antibodies against β-actin, HO1, NQO1 (all from Abcam), and NOX4 (Servicebio, Wuhan, China) at 4°C overnight. The membranes were then washed in TBST followed by incubation with goat anti-rabbit/mouse and horseradish peroxidase (HRP)-linked secondary antibodies, and the bands were detected using an enhanced chemiluminescence substrate (Tanon, Shanghai, China). The intensity of each band was determined using IPP software and was corrected to the intensity of β-actin.

### Immunofluorescence

Immunofluorescence analysis was performed with the following steps: after citrate antigen was repaired, the lung sections were blocked with 10% bovine serum albumin (BSA), subjected to incubation at 4°C overnight with primary antibodies. On the second day, the slides were incubated with the fluorescent secondary antibody at 37°C for 1 h, and then the sections were restrained with 4′,6′-diamino-2-phenylindole (DAPI) for nuclear location. The slides were observed under an upright fluorescence microscope (Leica, Germany).

### Fecal microbiome analysis

Total fecal DNA was extracted, and 1% agarose gel electrophoresis was performed to check the extracted genomic DNA. The 16S rRNA gene V3–V4 region was amplified with the following primers (forward primer, 5′-ACT​CCT​ACG​GGA​GGC​AGC​AG-3′; reverse primer, 5′-GGACTACHVGGGTWTCTAAT-3′) by polymerase chain reaction (PCR) (ABI 3 GeneAmp^®^ 9700, ABI, United States) before using QuantiFluor™-ST (Promega, United States) to quantify the PCR products, which were paired-end sequenced on an Illumina MiSeq instrument (Illumina, United States).

### Bioinformatics and statistical analysis

Operational taxonomic units (OTUs) were divided according to different similarity levels, and bio-information from OTUs with a similarity level >97% was analyzed using the RDP classifier (V2.13) and compared with the SILVA database (V138). In the diversity analysis of samples, alpha diversity reflects the richness and diversity of the microbial community, and mothur (V1.30.2) was used to analyze the species richness and diversity of the intestinal fora. R software was employed to create curves and analyze PCA graphics. LEfSe analysis was used to analyze the correlation between OTU and gate-level annotation.

### Untargeted metabolomics

Feces were collected from the rectums of mice into tubes with sterile scissors and tweezers, stored at −80°C, and then analyzed using a liquid chromatography–tandem mass spectrometry (LC–MS) system according to machine orders. Information from LC–MS was matched with metabolic public databases HMDB (http://www.hmdb.ca/) and KEGG (http://www.genome.jp/kegg/compound/) to obtain the classification of the metabolites. The annotated metabolites were then mapped to the KEGG pathway database (http://www.kegg.jp/kegg/pathway.html), and groups of differential metabolites were analyzed. Differential metabolite screening was performed in both groups with VIP (VIP >1) and *p*-value (*p* < 0.05).

### Statistical analysis

SPSS v.25.0 statistical software (IBM Corporation, USA) was used for statistical analyses. All data are represented as mean ± SEM. All assays were performed in at least three independent experiments. The statistical significance was calculated by a one-way ANOVA, which was conducted to analyze quantitative data. Dunnett’s *post hoc* test was used after one-way ANOVA analysis, for which *p* < 0.05 was considered significant.

## Results

### BFHX alleviates lung emphysema structural change of COPD mice

**FIGURE 1:**
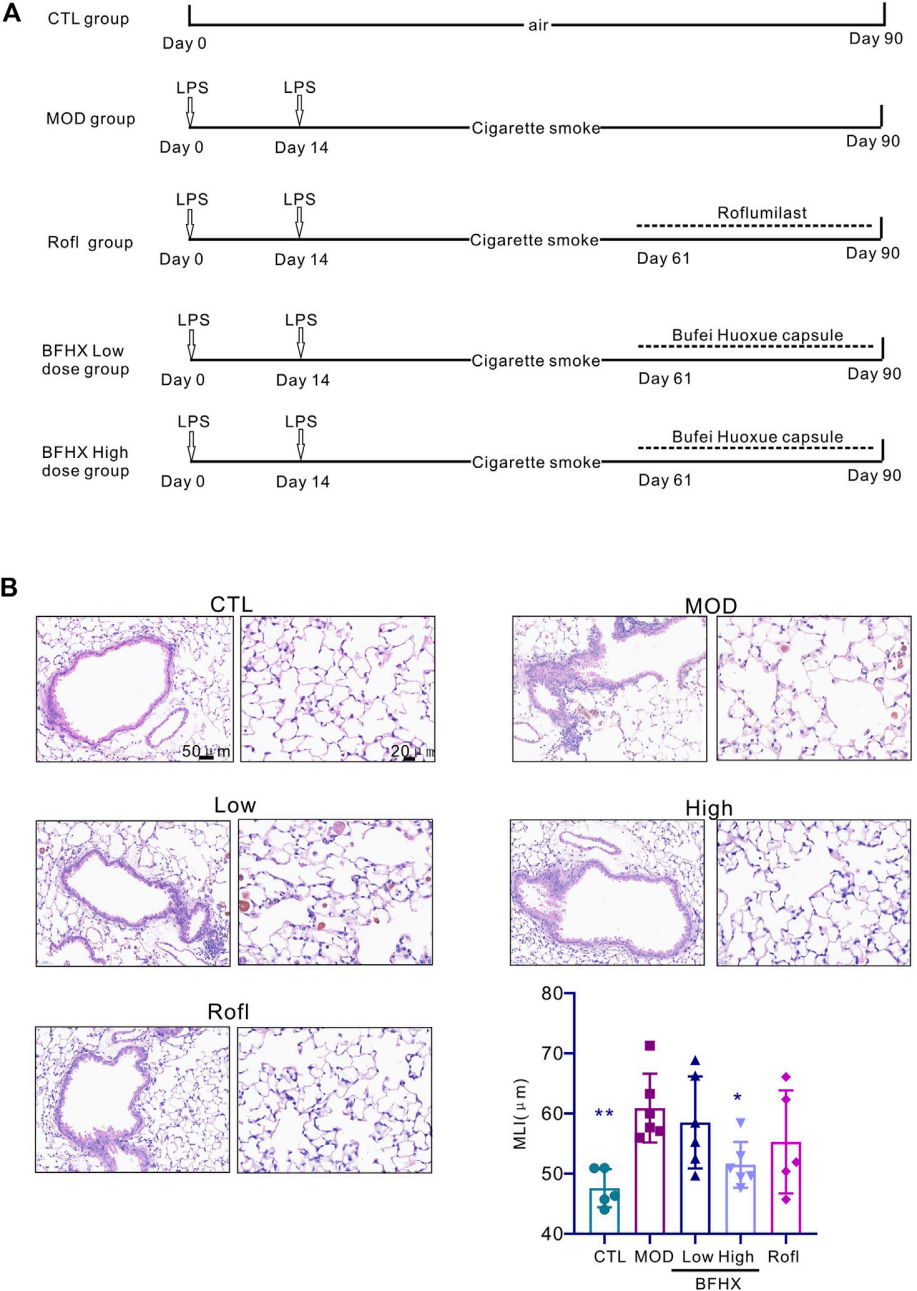
BFHX alleviates lung emphysema structural change of COPD mice. **(A)** Establishment of a COPD mouse model and treatment with BFHX. **(B)** The MLI and pathology of the mouse lung were assessed; BFHX: Bufei Huoxue capsule; results are shown as the mean ± SD, n = 4–6, vs. MOD group, *p < 0.05, and **p < 0.01.

During the experiment, the status of mice was observed, and the weight of mice was recorded. CTL group mice had normal movement, stable breathing, and shiny hair, whereas CS exposure mice had decreased activity, rapid respiration, and ruffled hair. Compared with the CTL group, CS exposure mice had slowed weight gain, and after the administration, there was no significant increase in weight gain (Supplementary Figure S1). To determine whether BFHX is harmful to other systems, the organ index (liver, spleen, and kidney) was tested. The results revealed no significant difference between the MOD and BFHX groups (Supplementary Figure S2). Airway inflammation and emphysema are the main pathological characteristics of COPD. Mice exposed to CS exposure displayed inflammation accumulation, destroyed alveoli, and increased MLI in lung tissue compared with the MOD group. The BFHX low and high groups showed reduced MLIs, indicating that BFHX reduced CS-induced emphysema (Figure 1).

### BFHX improves COPD mice pulmonary function

**FIGURE 2:**
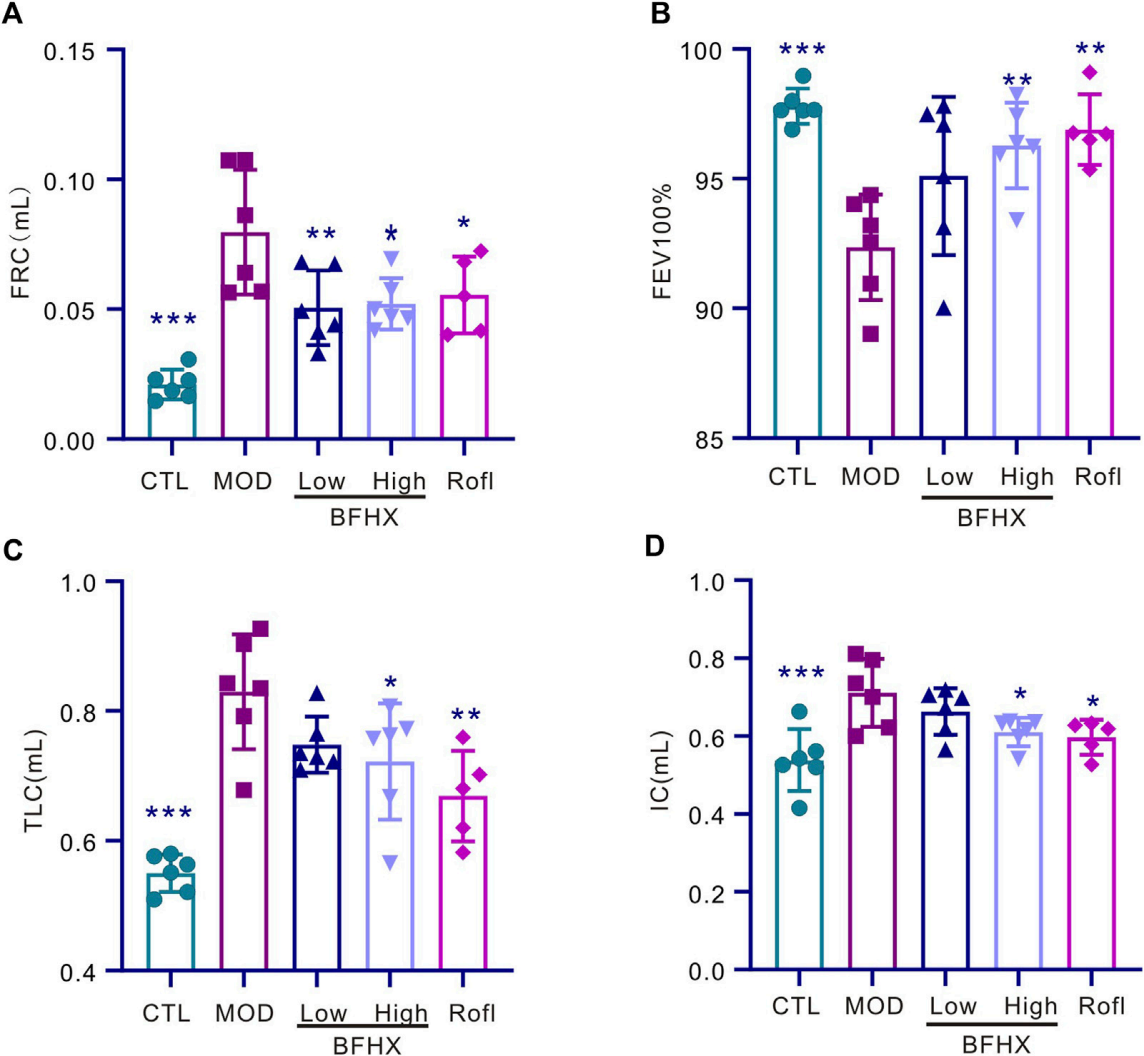
BFHX improves the pulmonary function of COPD mice. **(A–D)** The pulmonary function of mice, including FRC, FEV100%, TLC, and IC, was measured; FRC: functional residual capacity, FEV100%: percentage of forced vital capacity in 100 milliseconds (FEV100%), TLC: total lung capacity, IC: inspiratory capacity; results are shown as the mean ± SD, n = 5–6, vs. MOD group, *p < 0.05, **p < 0.01, and ***p < 0.001.

To determine the effect of BFHX on COPD mice, we performed pulmonary function testing and pulmonary function test after administration. As shown in Figures 2A–D, CS exposure significantly increased the functional residual capacity (FRC), percentage of forced vital capacity in 100 milliseconds (FEV100%), total lung capacity (TLC), and inspiratory capacity (IC) in the CS group. These results indicate that BFHX significantly improved the pulmonary function of COPD mice.

### BFHX reduces CS-induced lung inflammation

**FIGURE 3:**
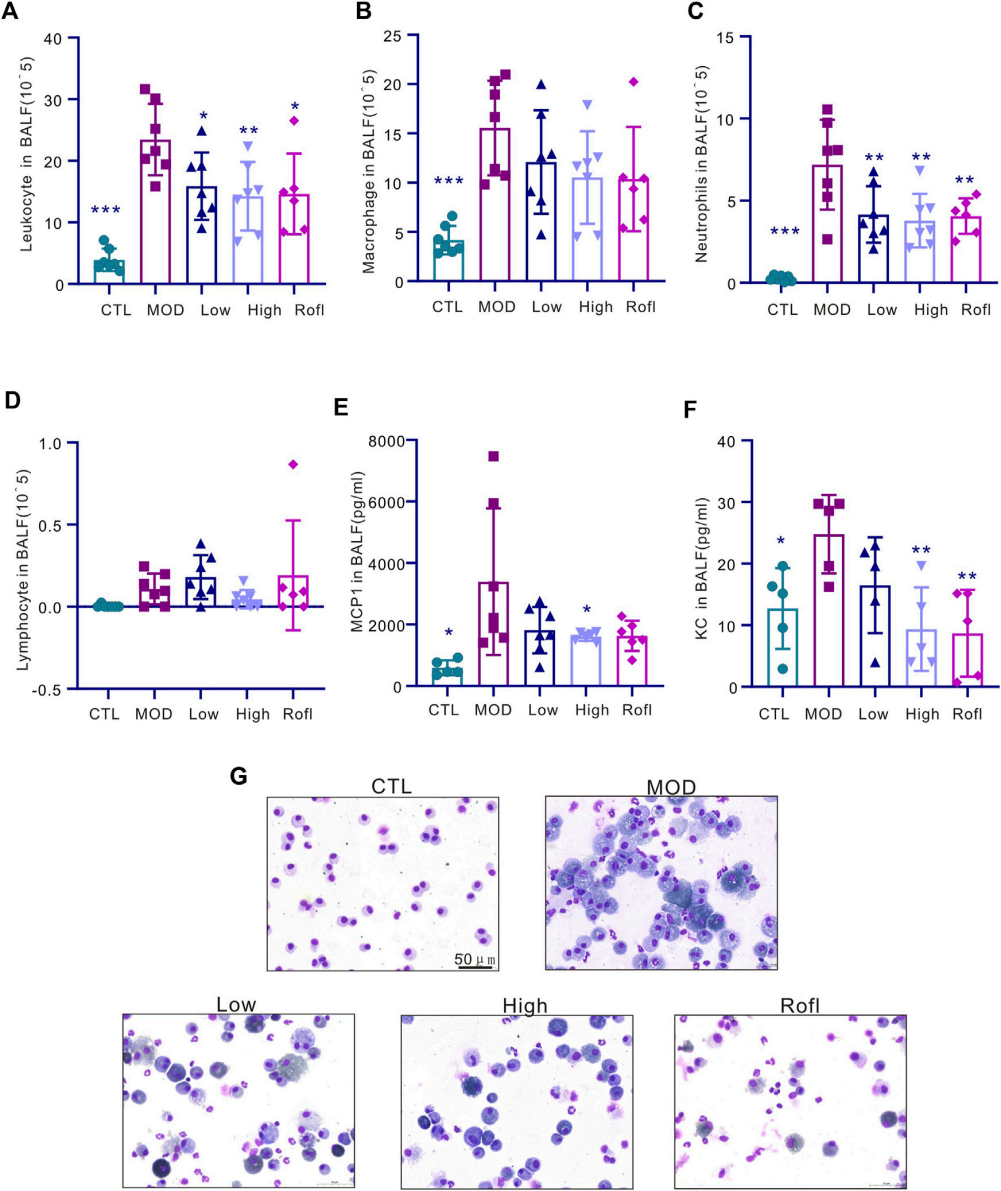
BFHX reduces CS-induced lung inflammation. **(A–D)** The total cell count and classification (macrophages, neutrophils, and lymphocytes) in BALF. **(E)** MCP1 level in BALF. **(F)** KC level in BALF. **(G)** Diagram showing cell morphologies in BALF; results are shown as the mean ± SD, n = 6–7, vs. MOD group, *p < 0.05, **p < 0.01, and ***p < 0.001.

CS induced accumulation inflammation in the cells of the lung, especially macrophages, which are the major cells in lung tissues. Acute inflammation induces acute exacerbation and promotes the progression of COPD. Consistent with the report (Arora et al., 2018), in our study, CS exposure induced inflammation accumulation in BALF (Figure 3A), predominantly macrophages (Figure 3B), whereas BFHX decreased the number of total inflammatory cells and cytokines (Figures 3E, F), indicating that BFHX suppressed CS-induced inflammation.

### BFHX inhibits CS-induced airway goblet cell hyperplasia

**FIGURE 4:**
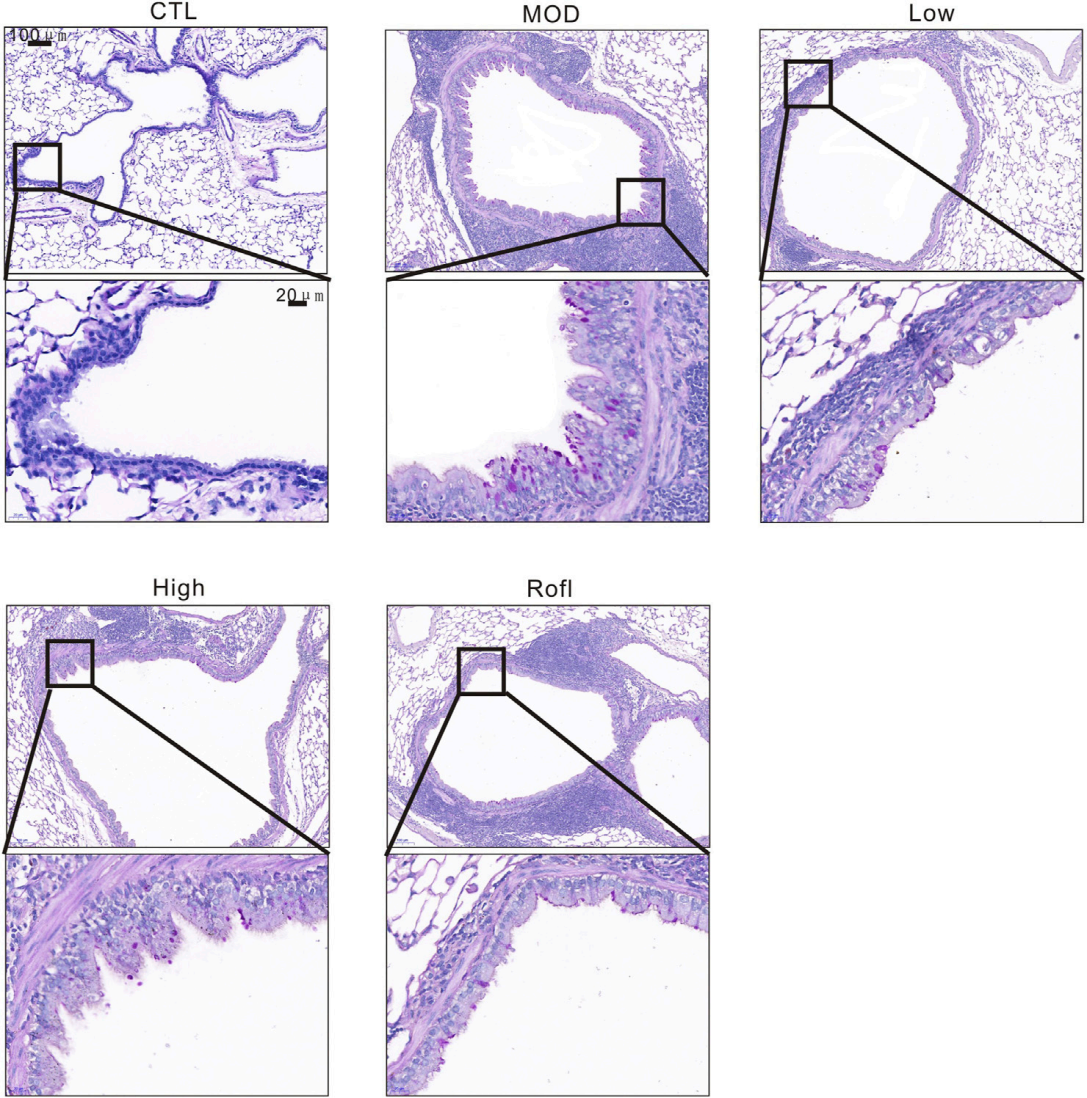
BFHX inhibits CS-induced airway goblet cell hyperplasia. PAS staining of a COPD mouse lung section.

As mucus promotes COPD progression and airway goblet cells produce mucus with CS exposure, we next evaluated the airway goblet cell hyperplasia of COPD mice. As shown in Figure 4, PAS staining of lung tissue revealed increased and decreased goblet cells in the airways of the MOD and BFHX groups, respectively.

### BFHX capsule decreases airway collagen deposition of COPD mice

**FIGURE 5:**
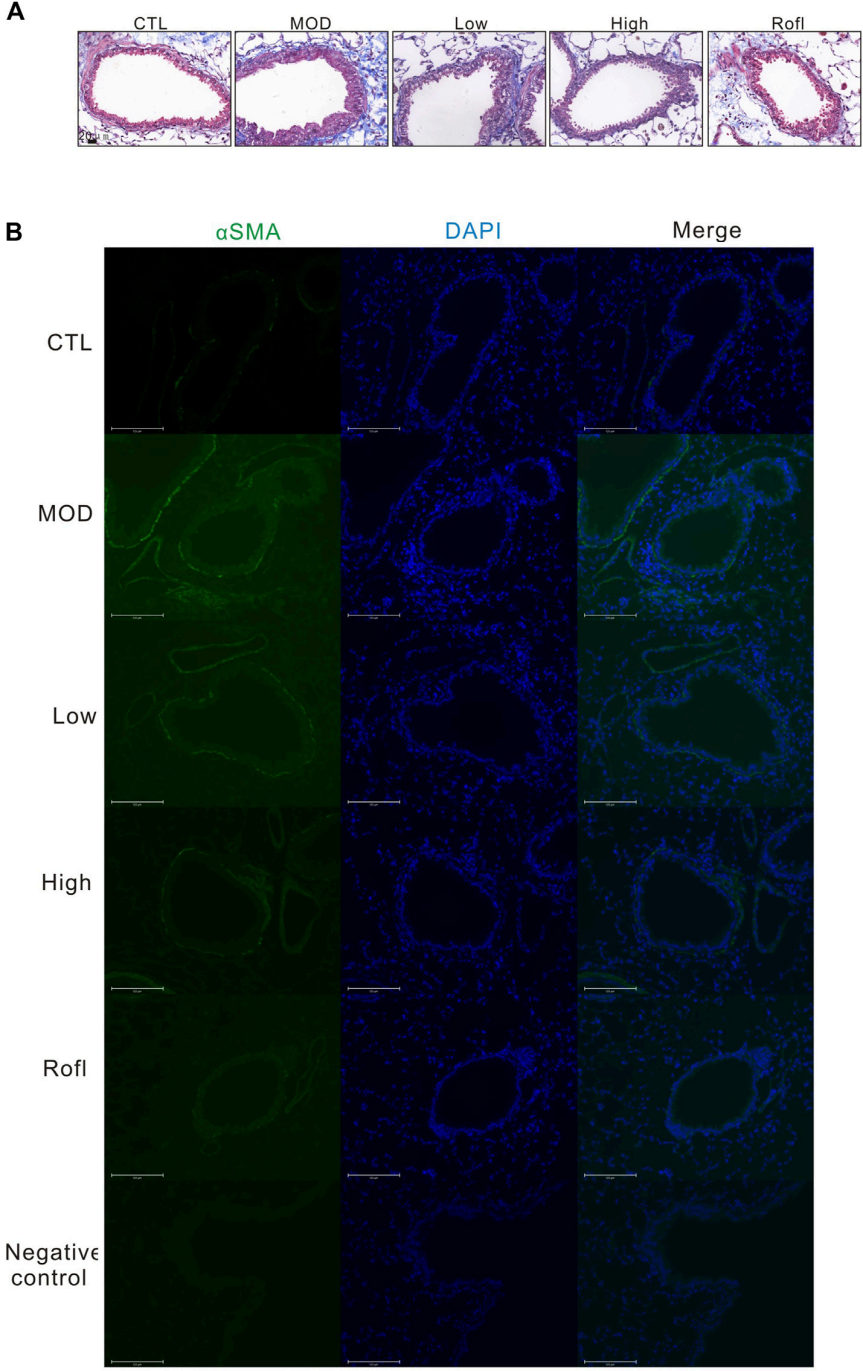
BFHX capsule decreases airway collagen deposition of COPD mice. **(A)** Masson’s staining of a COPD mouse lung section. **(B)** The αSMA level of the mouse airway.

Collagen deposition induces airway obstruction, which is a pathological characteristic of COPD. Therefore, we next conducted Masson’s staining of lung tissue to access collagen deposition. As shown in Figure 5, BFHX decreased collagen deposition in the airways of COPD mice. We also investigated the level of αSMA in the airways of COPD mice, as shown in Figure 5B. BFHX reduced the level of αSMA induced by CS.

### BFHX suppresses COPD-related oxidative stress responsive proteins *in vivo*

**FIGURE 6:**
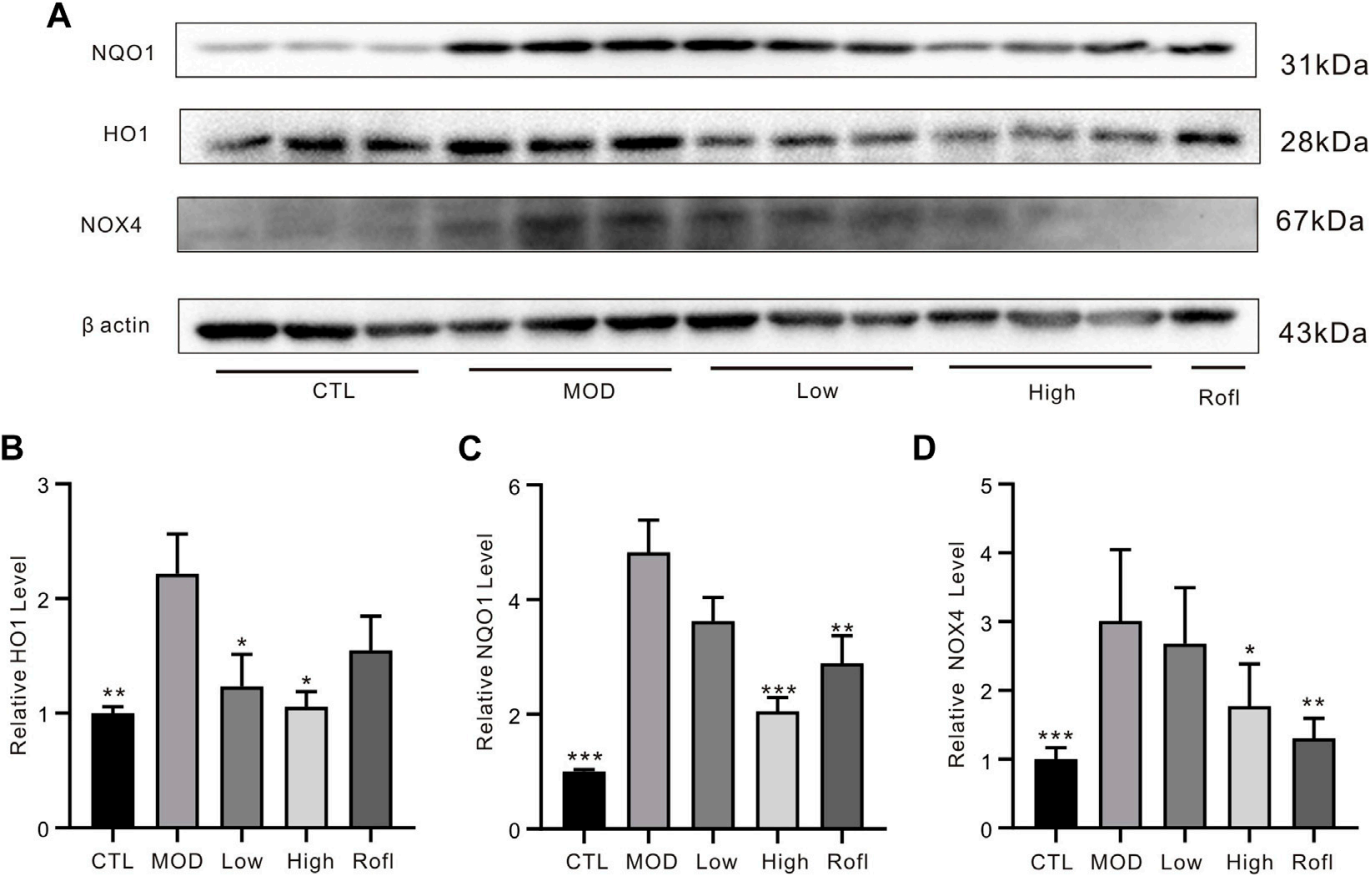
BFHX suppresses COPD-related oxidative stress responsive proteins *in vivo*. **(A)** WB bands of lung tissue. **(B)** WB band statistical results. Results are shown as the mean ± SD, n = 5, vs. MOD group, *p < 0.05, **p < 0.01, and ***p < 0.001.

CS exposure can cause oxidative stress accumulation and induce chronic lung inflammation. Excessive reactive oxygen species (ROS) from CS-induced oxidative stress accelerates lung inflammation and injury in COPD, both of which produce endogenous ROS from cellular sources such as dihydronicotinamide-adenine dinucleotide phosphate (NADPH) oxidase (NOX). As shown in Figure 6, BFHX decreased the level of NOX4 induced by CS exposure and LPS. In addition, the levels of antioxidant enzymes and phase 2 detoxifying enzymes, such as NADP(H): quinone oxidoreductase (NQO1) and the stress response protein heme oxygenase (HO-1), were increased in COPD mice and were found to be downregulated by BFHX.

### Overall structural changes in the microbiota composition

**FIGURE 7:**
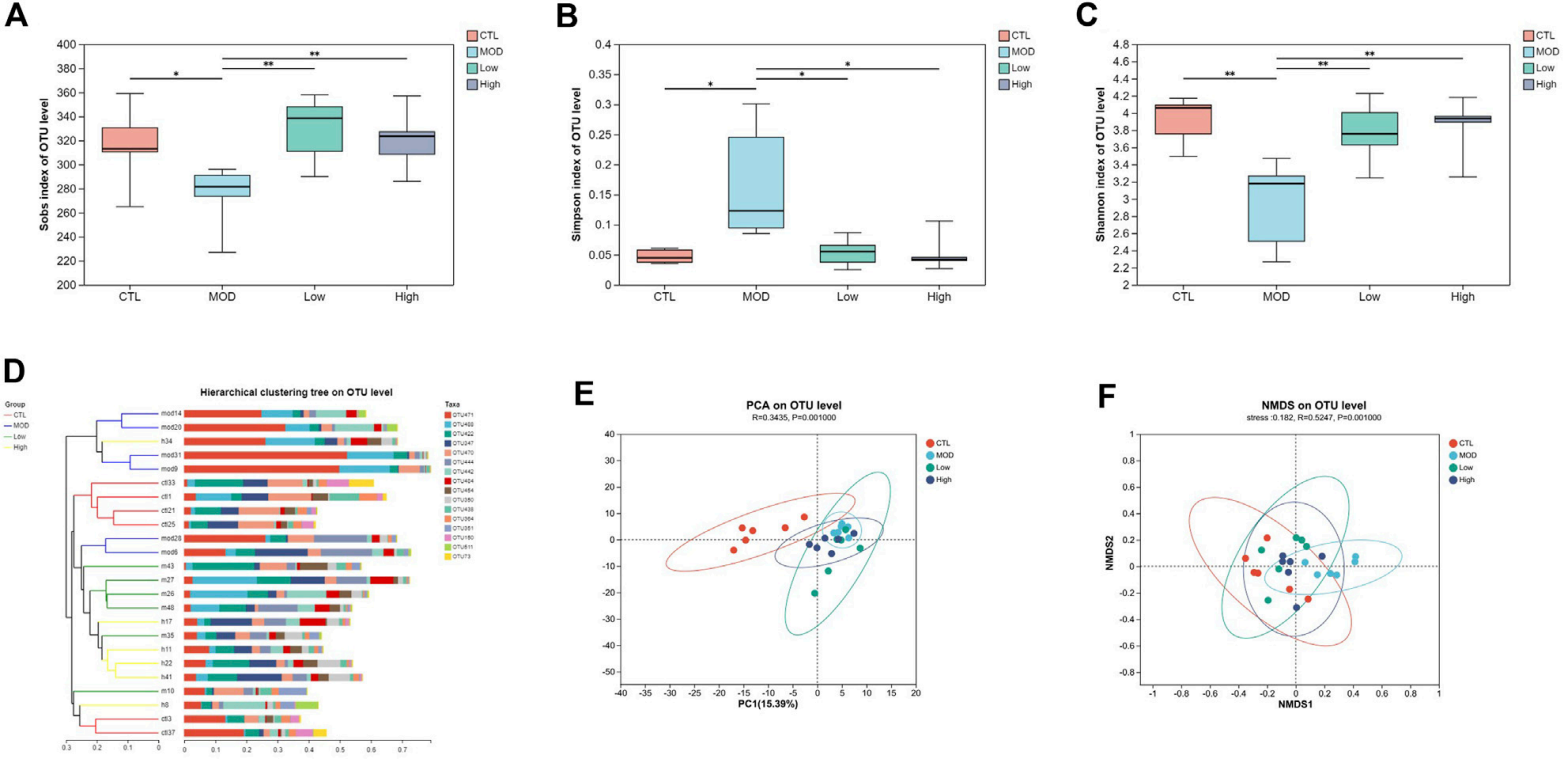
Overall structural changes in microbiota composition. **(A–C)** The Sobs, Simpson, and Shannon indices of each group. **(D)** Cluster based on the analysis of hierarchical clustering. **(E)** PCoA plots. **(F)** NMDS simplifies research objects in multidimensional space to low-dimensional space for positioning, analysis, and classification. Analyzing similarity tests (ANOSIM) were used to calculate significant differences between groups vs. MOD group, *p < 0.05, and **p < 0.01.

Next, the diversity of mice was detected following the administration of BFHX. The obtained dilution curve gradually fattened, indicating a sufficient amount of sequencing data and demonstrating the rationality of the sequencing data and, indirectly, the species richness (Supplementary Figure S3). The good coverage of each group was >99.0%, indicating that the sequencing depth of the microbiome analysis meets the requirements (Supplementary Figure S4). The α diversity of the intestinal flora includes the richness and diversity of microbial communities; the Sobs index was used to evaluate the richness, and the Simpson and Shannon indices were used to evaluate the diversity of the intestinal flora of COPD mice. As shown in Figure 7A, the richness of flora in the MOD group was lower, but it was increased by the BFHX administration. Figures 7B, C shows that the diversity of the bacterial population (Simpson and Shannon indices) in the MOD group was significantly lower than that in the CTL group, whereas BFHX reversed both the diversity and richness.

The beta diversity of each group was calculated based on cluster tree analysis, principal component analysis (PCA), and non-metric multidimensional analysis (NMDS). The PCA and hierarchical clustering tree (Figure 7D) were used to explore the similarities and differences in intestinal flora composition in each group. The results showed that the bacterial structure of the CTL group was separated from that of the MOD group and the BFHX administration group was similar to that of the MOD group. Based on the first two PCA scores, the total variations accounted for 15.39% and 9.84%, suggesting that COPD may change the percentages of bacteria in intestinal flora, and BFHX could improve bacterial composition (Figure 7E). The NMDS of the bacterial community also showed that BFHX could improve bacterial percentage (Figure 7F).

### Effect of BFHX on flora microbial classification based on the comparison of phylum and genus levels

**FIGURE 8:**
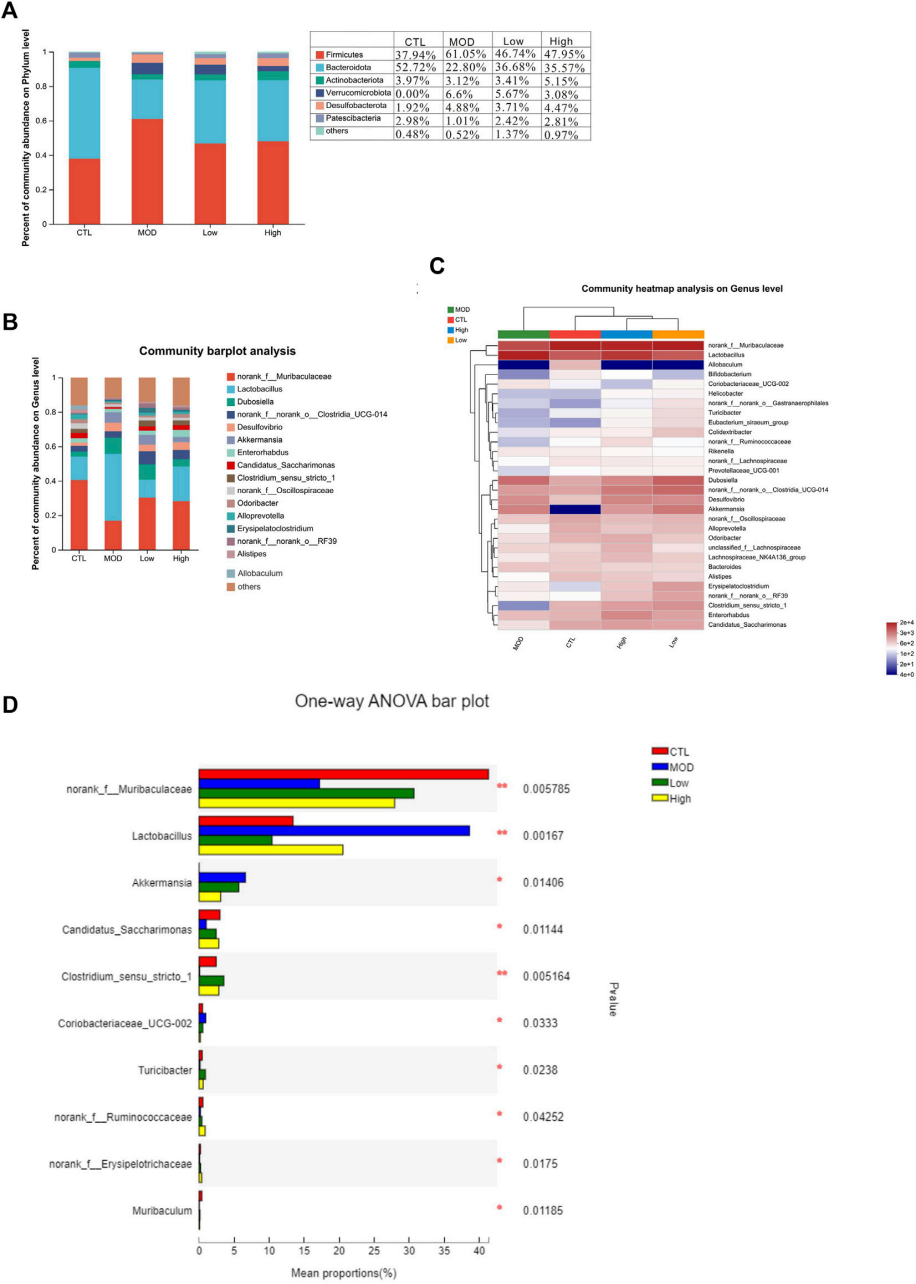
Effects of BFHX on microbial flora classification-based comparison of phylum and genus levels. *p < 0.05 and **p < 0.01. **(A,B)** Intestinal flora composition analysis at the phylum and genus levels. **(C)** Community heatmap. **(D)** The mean proportions of the top ten genus differences between each group.

Next, a heatmap was constructed based on the top 30 dominant genera using the SILVA database. The results shown in the heatmap revealed that all 30 genera belong to six main phyla: *Firmicutes*, *Bacteroidetes*, *Actinobacteria*, *Verrucomicrobiota*, *Desulfobacterota*, and *Proteobacteria*. At the phylum level, most of the dominant genera in the CTL group belonged to *Bacteroidetes*, and most of the dominant genera in the MOD group belonged to *Firmicutes* (Figure 8A). In addition, the proportion of *Verrucomicrobiota* and *Desulfobacterota* increased while that of *Actinobacteria* decreased in the MOD group compared with the CTL group. The results revealed that BFHX reversed the proportion of bacteria at the phylum level. At the genus level, the proportions of Murbaculaceae, *Lactobacillus*, *Desulfuribrio*, *Clostridiumthe*, and *Akkermansia* in the CTL group decreased sharply compared to those in the MOD group, whereas the proportions of *Lactobacillus*, *Desulfuribrio*, and *Bacteroidetes* increased substantially, indicating that BFHX reversed the proportions (Figures 8B–D).

### Effect of BFHX on dominant microbiota flora in COPD mice

**FIGURE 9:**
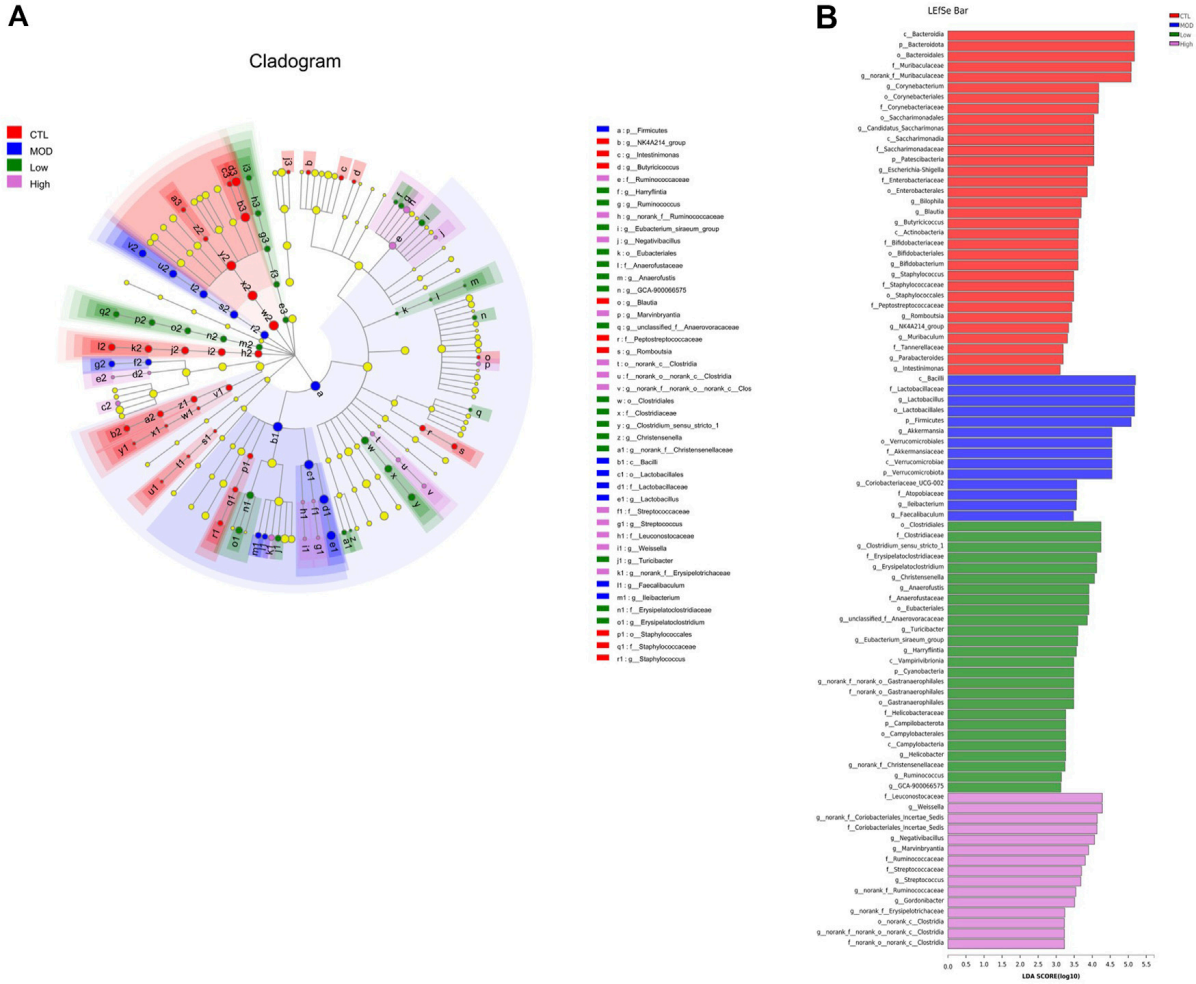
Effects of BFHX on the dominant members of the microbiota flora in COPD mice. **(A)** Colored nodes represent microbial groups that are significantly enriched in their corresponding groups, and the red nodes indicate a significant impact on the differences between groups. Pale yellow nodes indicate microbial groups that do not differ significantly across groups or have no significant effect on group differences. **(B)** Each group of bacteria with an LDA score of at least two.

To confirm the species differences at multiple levels, including the phylum, class, order, family, genus, and species levels, LEfSe was used to identify specialized communities in flora with LDA scores ≥2, as shown in Figure 9. From the phylum to the genus level, the number of dominant bacteria in each group were as follows: 33 in the CTL group, 14 in the MOD group, 26 in the low group, and 15 in the high group. In the CTL group, *Bacteroidota* dominated, whereas in the MOD, low, and high groups, *Firmicutes* dominated.

### Effect of BFHX on flora metabolites in COPD mice

**FIGURE 10:**
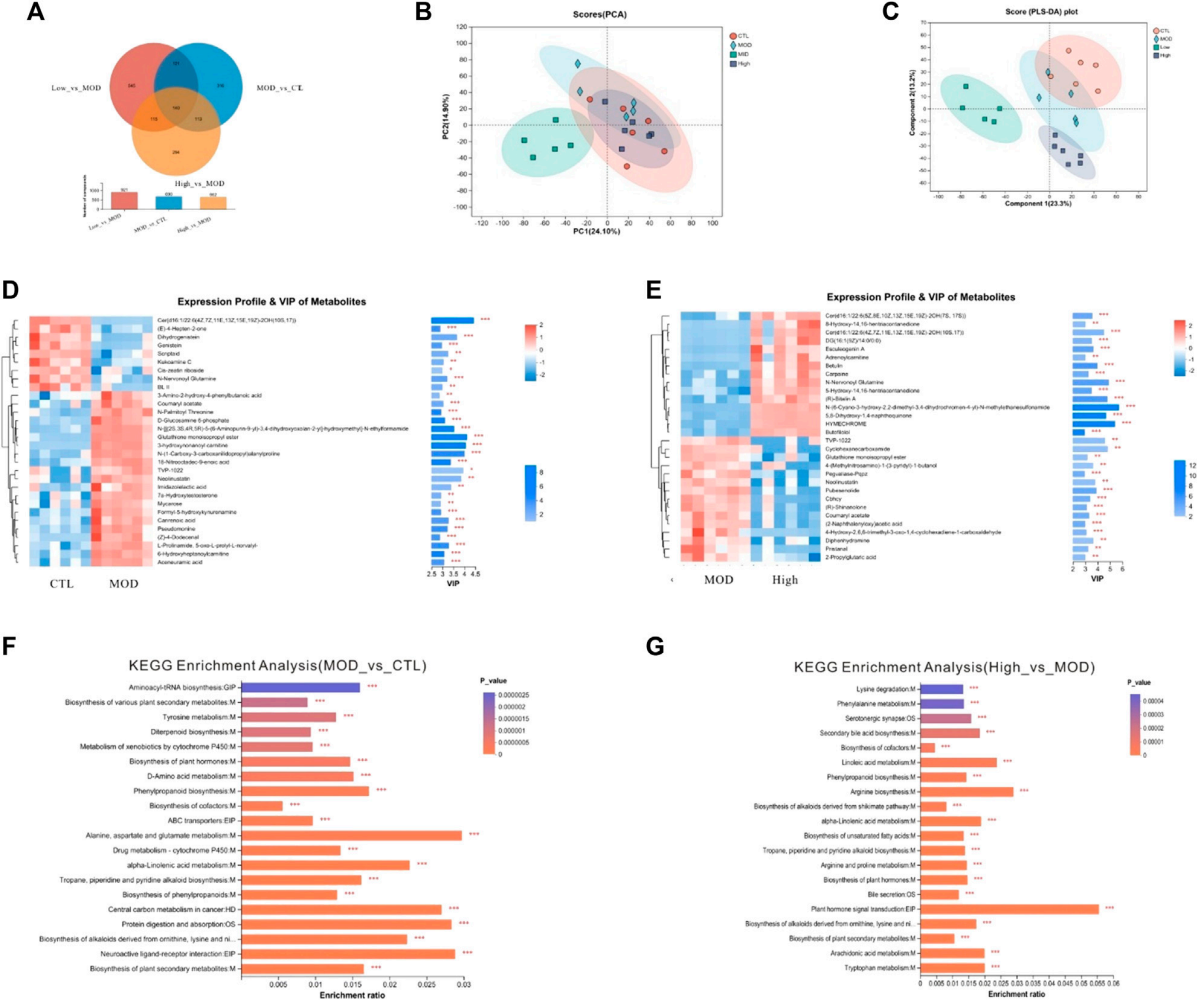
Effects of BFHX on flora metabolites in COPD mice. **(A)** Veen. **(B)** PCA analysis. **(C)** PLS-DA analysis. **(D,E)** clustering heatmap and VIP bar map of the different metabolites were compared between each of the two groups. **(F,G)** KEGG enrichment analysis between each of the two groups. *p < 0.05, **p < 0.01, and ***p < 0.001.

Next, non-targeted detection of mice fecal metabolites was performed based on LC–MS. As a result, 1784 fecal metabolites were identified in the CTL group, 1791 metabolites in the CS group, 1790 metabolites in the mid group, and 1794 metabolites in the high group. In addition, there were 17 metabolites in the CTL, mid, and high groups, and 20 metabolites in the MOD, mid, and high groups. According to different biological roles, all metabolites were classified as amino acids, vitamins and cofactors, nucleic acids, hormones and transmitters, carboxylic acids, antibiotics, steroids, organic acids, lipids, and carbohydrates. As shown in Figure 10B, PLS-DA analysis showed that the CTL, mid, and high groups’ data points were clearly separated from those of the MOD group. According to the PLS-DA analysis, the fecal metabolites of mice from the high group were much closer to those of the CTL group, and lysine degradation and phenylalanine metabolism were significantly altered. The differential metabolites between each group were screened by *p*-value and VIP bar graph (Figures 10D–F).

## Discussion

COPD, characterized by airflow limitation and airway obstruction, is a major public health burden. Indeed, the World Health Organization predicts that COPD will become the third leading cause of mortality worldwide in 2030 (Ahmadi et al., 2015). COPD is characterized by mucus hypersecretion and inflammation, both of which promote airway obstruction. Here, we established a mouse COPD model and orally administered BFHX capsule. We found that BFHX could improve COPD mice pulmonary function, suppress COPD-related lung inflammation, inhibit CS-induced oxidative *in vitro*, relieve CS-induced intestinal flora disorder, and regulate metabolites.

**FIGURE 11:**
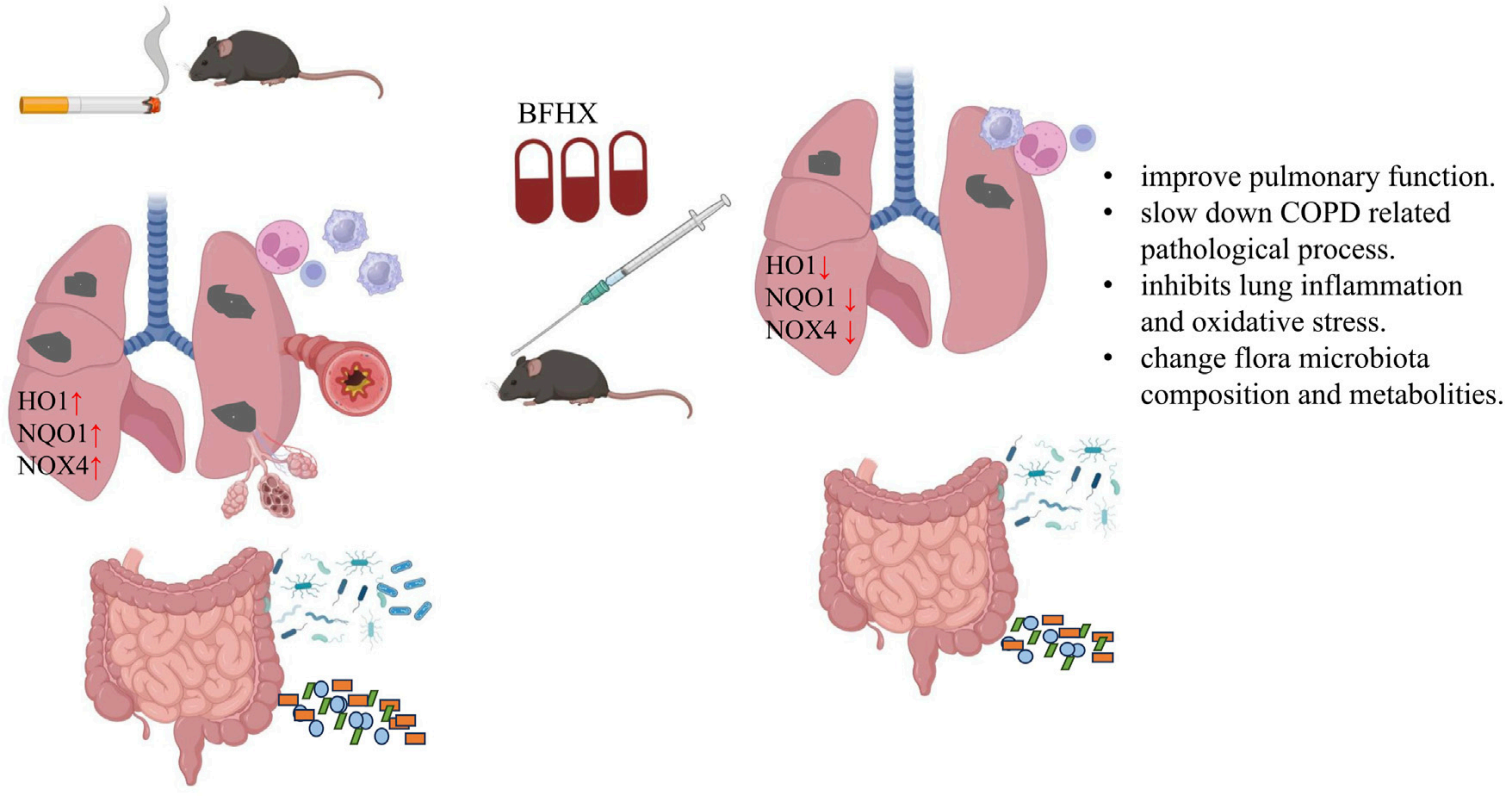
Bufei Huoxue capsule attenuates COPD-related inflammation and regulates intestinal microflora and metabolites. BFHX can improve mice pulmonary function, inhibit lung inflammation, and downregulate antioxidant (HO1, NQO1, and NOX4) levels of the lung. Moreover, BFHX can improve COPD-related flora microbiota composition and metabolite disorder.

The BFHX capsule consists of red peony, Psoraleae, and Astragalus. In addition to alleviating the oxidative stress and lung inflammation induced by chronic CS exposure *in vivo*, paeonol from red peony suppressed cigarette smoke extract |(CSE)-induced IL-8 *in vitro via* its antioxidant function and inhibition of the MAPKs/NF-κB signaling (Liu et al., 2014). Astragalus containing saponins, flavonoids, and polysaccharides has been used in folk medicine for its anti-inflammatory, immune stimulation, anti-oxidative properties (Ibrahim et al., 2013; Li et al., 2014). Psoraleae is a TCM which contains psoralea, coumarin, terpene phenolic compounds, flavonoids, *etc*. A growing body of research suggests that psoralea may have adequate effects in promoting bone formation and antioxidant and immune modulation activities (Wong and Rabie, 2011; Li et al., 2018) (Figure 11).

In a healthy state, there exists a delicate equilibrium between oxidants and antioxidants. However, the occurrence of hyperinflammatory injuries can disrupt this oxidative homeostasis, leading to the manifestation of various diseases (Victoni et al., 2021). Oxidative stress, resulting from an abundance of ROS or compromised endogenous antioxidant mechanisms, constitutes a primary contributing element in the development of COPD (Racanelli et al., 2018). CS induces increased oxidative stress, whereas oxidative stress causes inflammation and induces goblet cell metaplasia, producing mucus. In addition, CS is the main etiological risk factor involved in the pathogenesis of COPD. Furthermore, alongside the exogenous impacts of cigarettes, the human body generates an excessive amount of ROS during the progression of the disease (Schiffers et al., 2021; Liu et al., 2022). Consequently, oxidative stress may be a therapeutic target for patients with COPD and acute exacerbations (Barnes, 2020; Zhao and Wu, 2021). Based on the harmful effects of CS-induced oxidative stress, we checked the relative protein expression levels of HO1 and NQO1, which, compared with the CTL group, were higher following CS exposure. This excessive production of oxidative stress factors may contribute to the higher antioxidant capacity observed following exposure to harmful environments such as CS.

Under pathological conditions, there is a reciprocal influence between the gut and the lung, which occurs through the gut–lung axis (Keely and Hansbro, 2014; Budden et al., 2017a). The reciprocal relationship between lung disease and gastrointestinal health is evident (Keely et al., 2012). In the human microbiome, 99% of the bacteria live in the gut. Under physiological conditions, the bacteria maintain intestinal homeostasis; however, under pathological conditions, the microbiome can change to facilitate a disease-promoting environment (Gomaa, 2020). Bowerman et al. (2020) identified significant differences between 28 patients diagnosed with COPD and 29 healthy individuals. *Streptococcus* sp000187445, *Streptococcus* vestibularis, and various members of the Lachnospiraceae family exhibited a significant association with diminished pulmonary function (Bowerman et al., 2020).

CS leads to gut and lung microbiota disorders, which are associated with lung inflammation and immune responses (Sze et al., 2014; Saint-Criq et al., 2020; Bai et al., 2022). In addition, the gut microbiome regulates host immune responses to respiratory infection (Trompette et al., 2018) and may contribute to the frequency of exacerbations in COPD. In circulation, the chemicals in CS directly induce inflammation and microbiome dysbiosis (Ding et al., 2021; Lai et al., 2022). Four bacterial phyla, including *Firmicutes*, *Bacteroidetes*, *Actinobacteria*, and *Proteobacteria*, dominate the healthy gut (Mathieu et al., 2018). At the phylum level, *Firmicutes* and *Bacteroidetes* are the predominant bacterial taxa in the gastrointestinal tract (Trivedi and Barve, 2020), although the relative abundance can change with disease progression and disease treatment. For example, the abundance of *Firmicutes* has been shown to increase following the administration of chemotherapy (Zhang et al., 2020). Moreover, the proportion of *Bacteroidetes* and *Firmicutes* in the gut has been shown to be significantly different in chronic lung disease (Begley et al., 2018). Indeed, a study of 73 healthy control subjects and 99 patients with COPD showed that patients with COPD had a higher proportion of *Bacteroidetes* and a lower proportion of Firmicutes (Li et al., 2021), which is consistent with our findings. We found that the relative abundance of *Firmicutes* in the flora of COPD model mice was significantly higher than that in the normal group, whereas the relative abundance of *Bacteroides* was decreased. Many effective components of traditional Chinese medicine have exhibited useful biological activity and therapeutic effects on gut microflora (Yu et al., 2019; Mao et al., 2022).

Untargeted metabolites between MOD and high-dose groups were enriched in the lysine degradation and phenylalanine metabolism pathways, which is a pathway confined to the mitochondria which is ketogenic, yielding two acetyl-CoAs and reduction equivalents (Leandro and Houten, 2020). In the process of lysine degradation, cadaverine is formed. Cadaverine is the precursor of N-acetylcadaverine, and the latter has previously been associated with Crohn’s disease (Diederen et al., 2020). Gut bacteria contribute to anti-inflammation in patients with multiple sclerosis *via* phenylalanine metabolism (Shrode et al., 2022). The deregulated tyrosine–phenylalanine axis in the urine may be a potential target for diagnostics and intervention in tuberculosis patients (Das et al., 2015). BFHX play a role in anti-inflammation and anti-oxidant effect of COPD by regulating lysine degradation and phenylalanine metabolism pathway. This study has some limitations that warrant discussion. First, we found no obvious dose–effect relationship, possibly due to improper dosage setting; the dose (1.9–3.8 g/kg/day) may be given at an ineffective interval, and the composition of BFHX that contributes to the effect is unknown. Second, although BFHX was found to improve the flora disorder, the mechanism and interaction between the effective bacteria and metabolites have not yet been explored.

In conclusion, BFHX protects mice from CS-induced lung inflammation and oxidative stress by regulating intestinal flora disorder and metabolites.

## Data Availability

The raw 16S rRNA sequencing data presented in the study are deposited in the NCBI BioProject, the accession number is PRJNA1073266.
