# Supplementary figures and images for: Bufei Huoxue capsule attenuates COPD-related inflammation and regulates intestinal microflora, metabolites
Source: Front Pharmacol. 2024 Apr 9;15:1270661. doi: 10.3389/fphar.2024.1270661 (PMC11041376; doi:10.3389/fphar.2024.1270661)

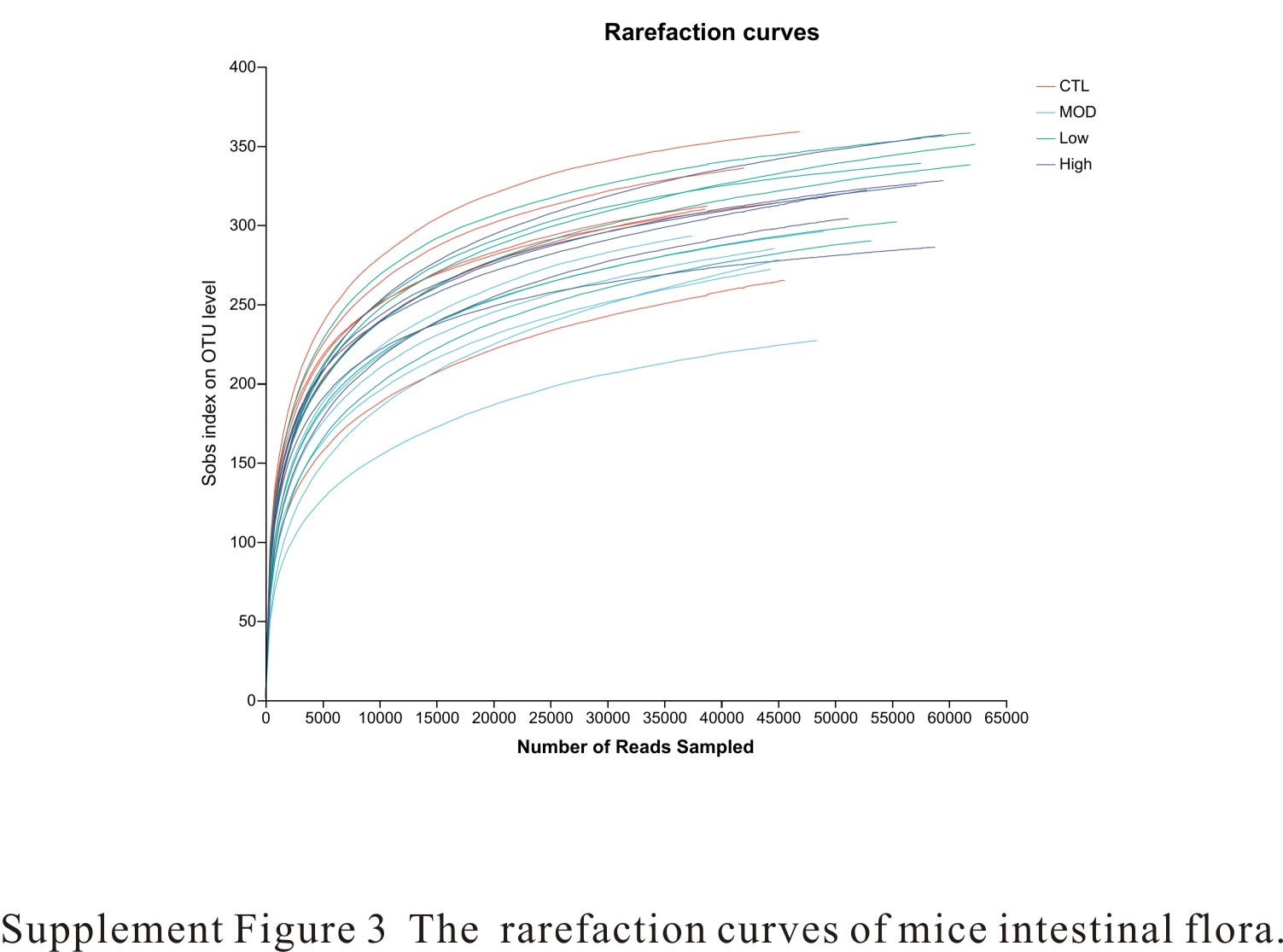

Supplement: Supplementary file 1 [file Image3.JPEG]

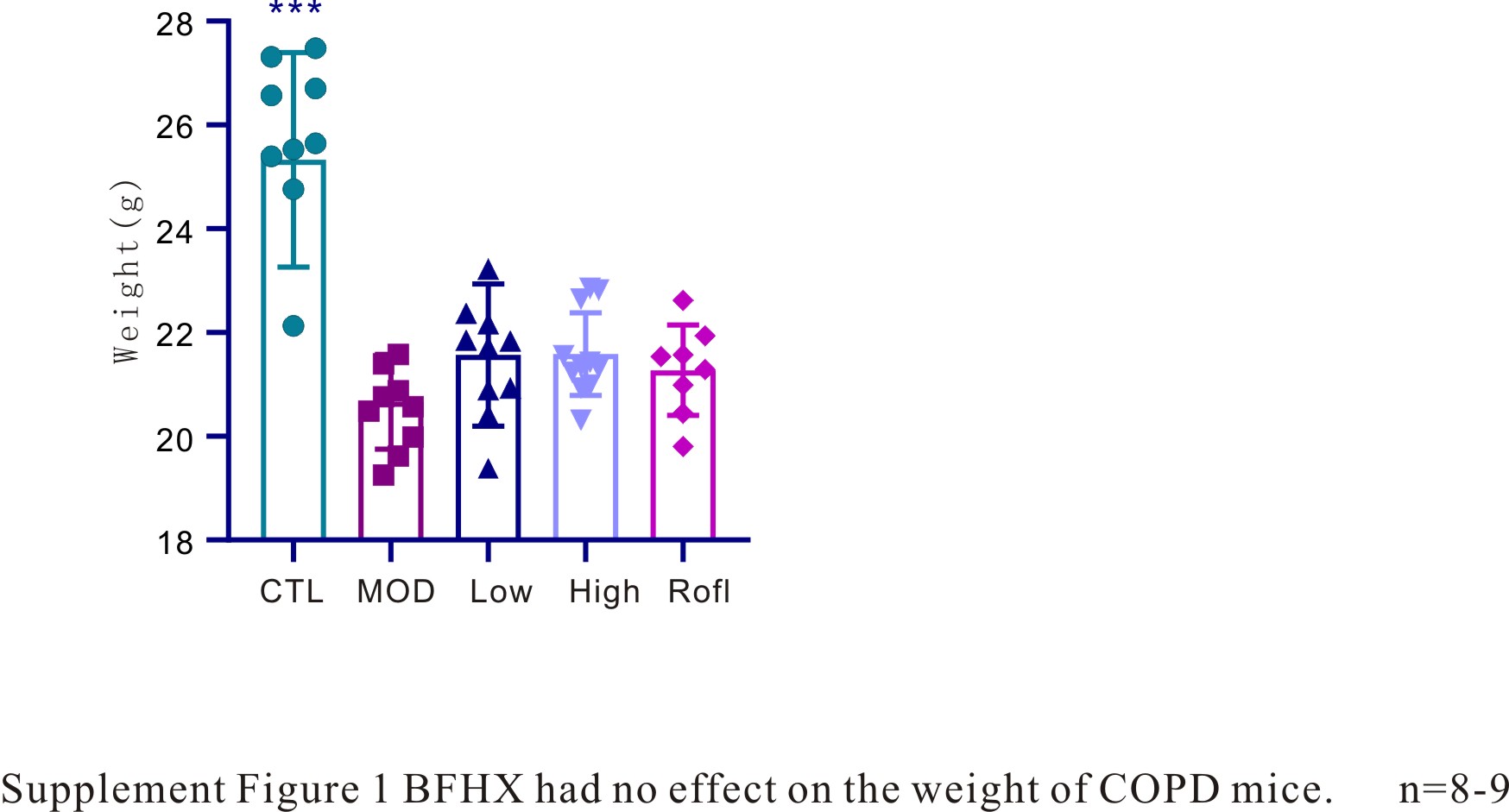

Supplement: Supplementary file 2 [file Image1.JPEG]

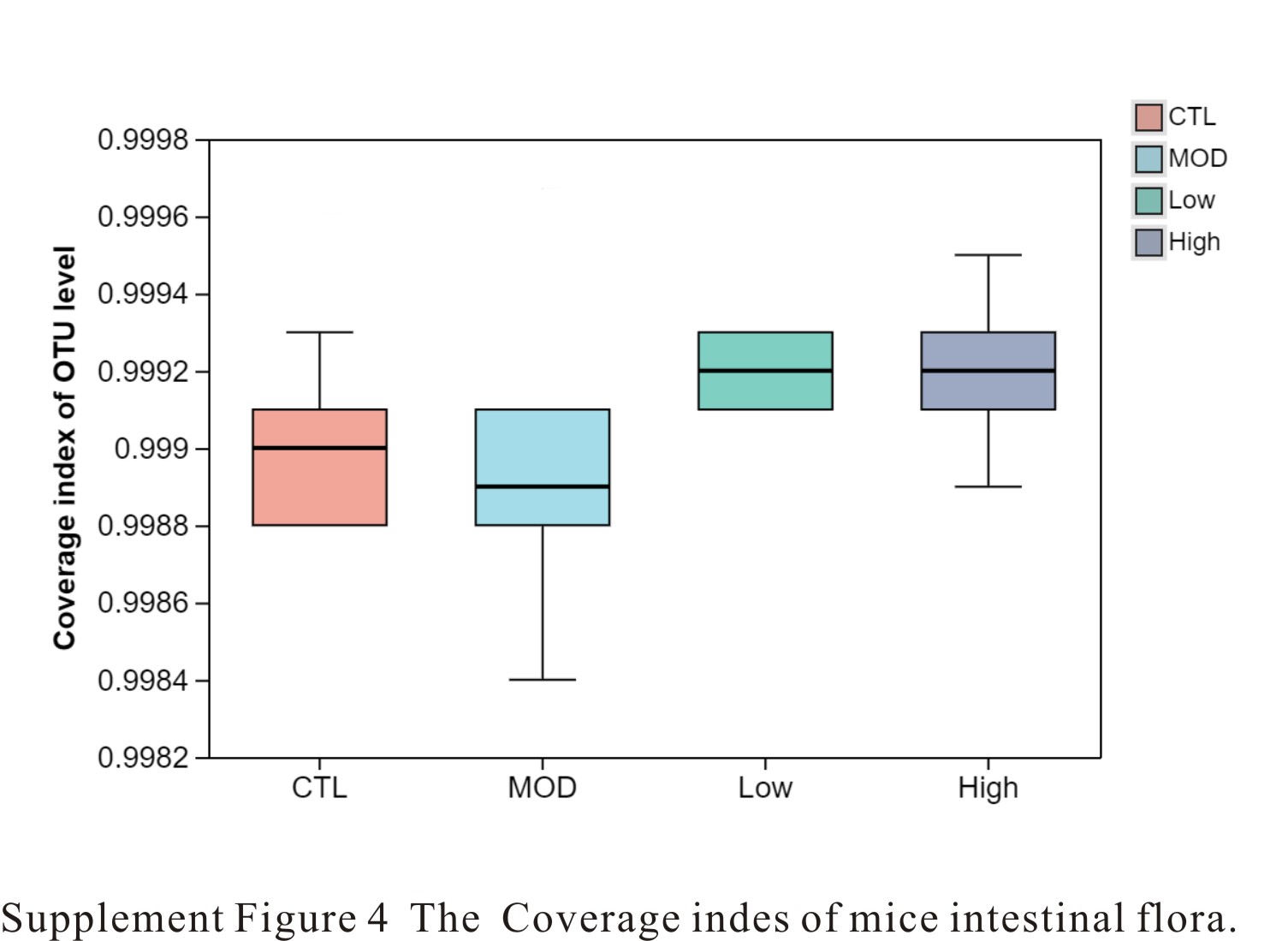

Supplement: Supplementary file 3 [file Image4.JPEG]

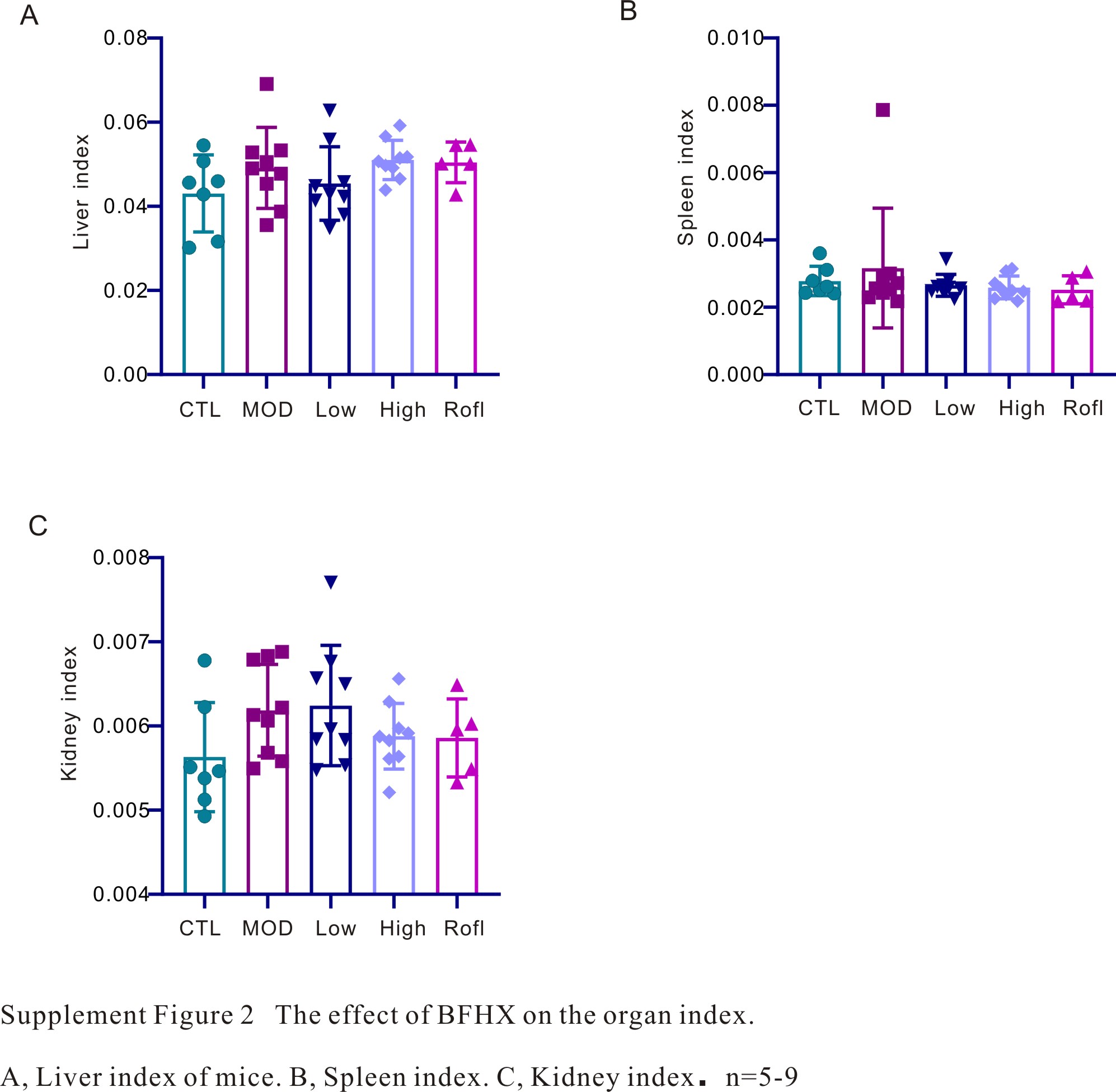

Supplement: Supplementary file 4 [file Image2.JPEG]

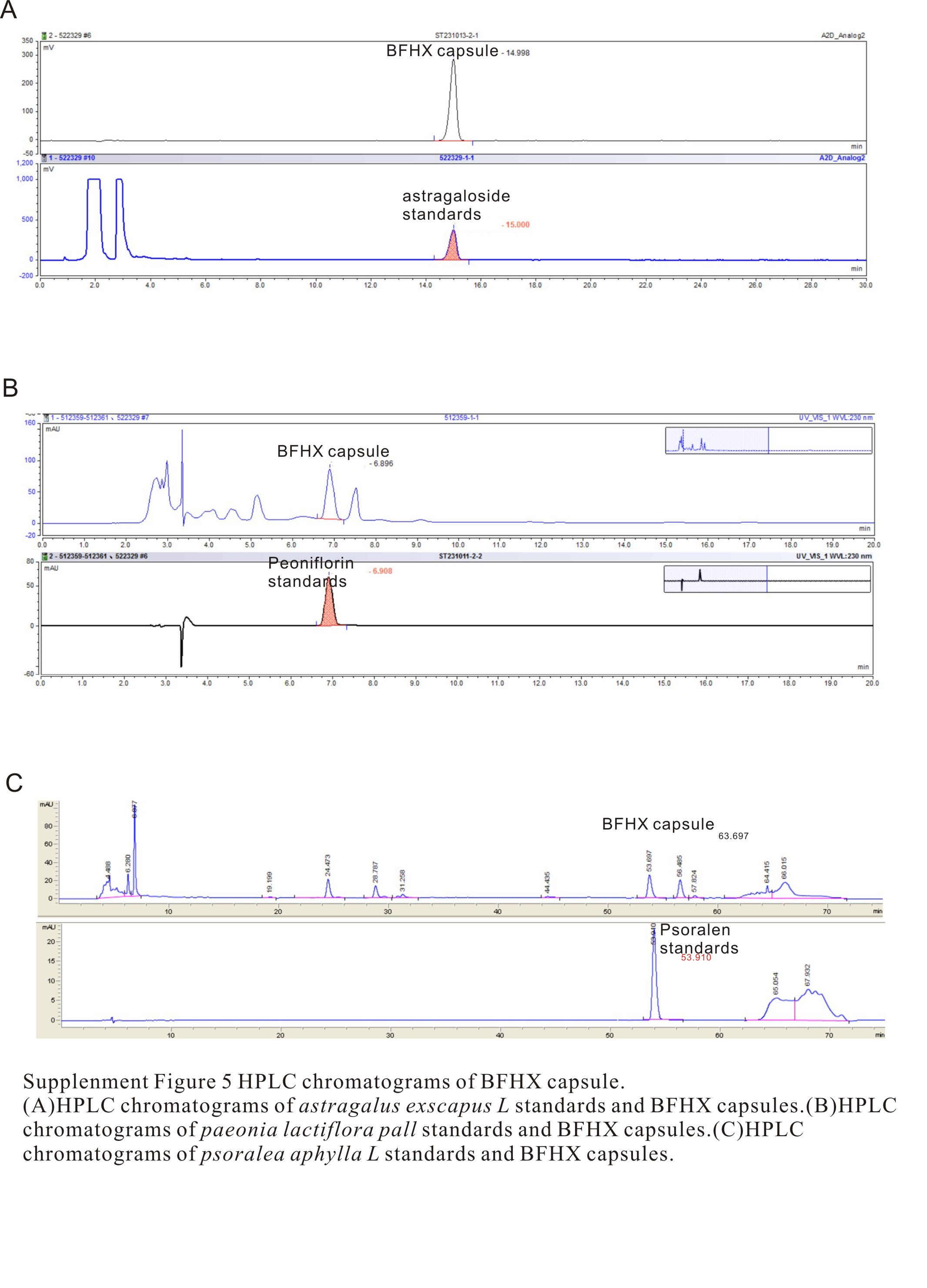

Supplement: Supplementary file 5 [file Image5.JPEG]
